# Occurrence, types and distribution of calcium oxalate crystals in leaves and stems of some species of poisonous plants

**DOI:** 10.1186/1999-3110-55-32

**Published:** 2014-03-15

**Authors:** Sevil Tütüncü Konyar, Necla Öztürk, Feruzan Dane

**Affiliations:** Faculty of Science, Department of Biology, Trakya University, Edirne, 22030 Turkey

**Keywords:** European Turkey, Poisonous plants, Ca-oxalate crystals, Druse crystals, Crystal sands, Prismatic crystals

## Abstract

**Background:**

Calcium oxalate crystals, which are found in many organs of plants, have different morphological forms: as druses, prism, styloids, raphides and crystal sand. In this study, the distribution, type and specific location of calcium oxalate crystals in the leaves and stems of the eight species of poisonous plants and one species of nonpoisonous plant were investigated with light microscopy. During study special attention was given to the possible correlation between the presence and types of calcium oxalate crystals and toxic plant organs. The plants examined in this study were *Hedera helix* L. (Araliaceae), *Aristolochia clematitis* L. (Aristolochiaceae), *Humulus lupulus* L. (Cannabaceae), *Saponaria officinalis* L. (Caryophyllaceae), *Chelidonium majus* L. (Papaveraceae), *Hypericum perforatum* L. (Hypericaceae), *Tribulus terrestris* L. (Zygophyllaceae), *Cynanchum acutum* L. (Asclepiadaceae), and *Nerium oleander* L. (Apocynaceae).

**Results:**

Three types of crystals: druses, prismatic crystals and crystal sands were observed. Druses were identified in the leaves and stems of six species of studied plants. In contrast to druses, crystal sands and prismatic crystals were rare. Prismatic crystals were observed in the leaf mesophlly cells of both *Nerium oleander* and *Cynanchum acutum.* However, crystal sands were observed only in the pith tissue of *Humulus lupulus.* On the other hand, leaves and stems of *Chelidonium majus*, *Aristolochia clematitis* and *Hypericum perforatum* were devoid of crystals.

**Conclusion:**

There is no absolute correlation between the presence and type of calcium oxalate crystals and toxic plant organs. However druse crystals may function as main irritant in toxic organs of the plants.

**Electronic supplementary material:**

The online version of this article (doi:10.1186/1999-3110-55-32) contains supplementary material, which is available to authorized users.

## Background

Plants containing toxic substances in amounts that can cause illness or death of humans and animals are called poisonous plants (Aplin 1976). Depending on the plant species, the poisonous parts of the plants can be the root, rhizome, bulb, stem, branch, leaf, flower, fruit, seed, pollen, nectar or sap (Seçmen and Leblebici 1987). There are several toxicologically significant plant constituents such as oxalates, alkaloids, glycosides, amino acids, proteins, minerals, acids, terpenes, phytotoxins, photosensitizing compounds, phenolics and tannins. These are generally known as secondary metabolites (Frohne and Pfander 1984). In many plants, oxalates are metabolized very slowly or not. However, they might have a toxic effect when accumulating in excess quantities (Franceschi and Horner 1980). Moreover, free calcium at high concentrations is also toxic to cells. Therefore plants could induce calcium oxalate crystal formation to remove excess oxalate or calcium (Çalışkan 2000).

Calcium oxalate crystals occur in more than 215 higher plant families (McNair 1932 Franceschi and Horner 1980; Lersten and Horner 2006) including gymnosperms and angiosperms. In angiosperms crystal formation is generally intracellular and crystals form inside the vacuoles of specialized cells called idioblast. However, in gymnosperms most of the crystals form in the cell wall (Kinzel 1989). Crystal formation in idioblasts is usually related with membranes, chambers, or inclusions found within the vacuoles. Crystal idioblasts have different shapes, sizes and intracellular structures than non-crystal- forming cells of the same tissue (Horner and Wagner 1995) and may also contain, tubules modified plastids and enlarged nuclei (Franceschi and Horner 1980). Additionally, the idioblasts undergo ultrastructural modifications depending on crystal precipitation.

Although the shape, size and number of crystals show variations among taxa, they have been classified into five main groups based on their morphology: as prism, druses, styloids, raphides and crystal sand (Webb 1999). Various physical, chemical and biological parameters such as light, temperature, pH, ion concentration and herbivory may affect the location, size and other properties of crystals in plants (Franceschi and Horner 1980; Molano-Flores 2001; Kuo-Huang et al. 2007; Meriç 2009). However, many authors have stated that crystal formation within the cell is under genetic control (Ilarslan et al. 2001). Thus the shape and location of the crystals within a taxon are often very specific and may be represented as a taxonomic character (Genua and Hillson 1985; Prychid and Rudall 1999; Lersten and Horner 2000). Furthermore, the presence or absence of crystals may represent useful taxonomic characters and also be used for understanding the evolutionary relationships of plant species (Franceschi and Horner 1980; Prychid and Rudall 1999). For example, by considering both morphological and molecular characteristics and the distribution of calcium oxalate crystals in some taxa, Rudall and Chase (1996) have shown that the genera formerly included in Xanthorrhoeaceae *sensu lato* may belong to three different families: Xanthorrhoeaceae *sensu stricto*, Lomandraceae and Dasypogonaceae (Prychid and Rudall 1999).

Calcium oxalate crystals may be present in almost all parts of the plant. Presence of calcium oxalate crystals have been reported in roots (Horner et al. 2000; Dane et al. 2000; Aybeke 2012a), leaves (Horner and Whitmoyer 1972; Doaigey 1991; Faheed et al. 2013), stems (Grimson and Arnott 1983; Meriç 2008,2009; Aybeke et al. 2010), seeds (Buttrose and Lott 1978; Lott and Buttrose 1978; Webb and Arnott 1982,1983; Ilarslan et al. 2001; Meriç 2008,2009), floral organs (Tilton and Horner 1980; Meriç and Dane 2004; Ekici and Dane 2007) and anthers (Horner and Wagner 1980;1992; Ekici and Dane 2009; Aybeke 2012b), and root nodules (Sutherland and Sprent 1984). These crystals can be located in specific tissues such as epidermis, cortex, phloem, xylem and pith or they may be distributed all over the plant.

Many functions have been attributed to calcium oxalate crystals in plants such as participating in calcium homeostasis, storage of calcium (Franceschi 1989), removal of excess oxalate, metal detoxification, tissue support, light gathering and reflection (Franceschi and Horner 1980), and protection against insects and foraging animals. Calcium oxalate crystals protect plants against herbivores by their association with irritating chemicals or with proteolytic toxins (Rupali et al. 2012). Mechanical effect of needle like crystals which puncture the foraging animals is also important part of the plant defence.

Many forage plants can accumulate oxalate in toxic concentrations (Dhillon et al. 1971; Cheeke 1995; Rahman et al. 2006). Therefore most of the oxalate-containing plants may cause poisoning to ruminants. However, susceptibility of animals to oxalate poisoning depends on factors such as chemical form of the oxalate, age of the animal, adaptation of animals to oxalate-rich forage, composition of the diet and availability of water for animals (Rahman and Kawamura 2011).

Although there have been numerous studies on calcium oxalate crystals in plants, only a few studies (Fasset 1973; Doaigey 1991) have been carried out to investigate the relationship between calcium oxalate crystals and toxicity of plants. Therefore, in the present study, types and specific locations of calcium oxalate crystals in the stems and leaves of the eight species of poisonous plant and one species of non- poisonous plant were investigated to reveal the possible relationship between calcium oxalate crystals and poisonous organs of plants. Non- poisonous plant, *Humulus lupus* L., was examined in order to provide a comparative demonstration of the relationship between the poisonous properties of the plants and their crystals.

The primary aim of this study was (1) to contribute to the previous studies by testing the hypothesis which suggests the relationship between the presence of calcium oxalate crystals and the toxicity of plant organs, (2) to provide data for the taxonomic and phylogenetic studies by reporting the type and specific location of calcium oxalate crystals in selected species of plants.

## Methods

In this study, eight species of poisonous plants and one species of nonpoisonous plants belonging to different families were collected from natural habitats in Edirne Province (European Turkey).

Collected species were identified by Feruzan Dane. The voucher specimens were kept in the herbarium of Trakya University (EDTU). The collection data of the investigated specimens were given in Table 1.

**Table 1:** **List of examined species and their collection data**

Species	EDTU number	Collector	Date of collection	Location
*Aristolochia clematitis* L.	13385	Dane & Öztürk	16 July 2008	Söğütlük, Edirne
*Chelidonium majus* L.	13386	Dane & Öztürk	16 July 2008	Söğütlük, Edirne
*Cynanchum acutum* L.	13387	Dane & Öztürk	16 July 2008	Söğütlük, Edirne
*Hedera helix* L.	13388	Dane & Öztürk	16 July 2008	Söğütlük, Edirne
*Humulus lupulus* L.	13389	Dane & Öztürk	15 July 2008	Söğütlük, Edirne
*Hypericum perforatum* L.	13390	Dane & Öztürk	15 July 2008	Söğütlük, Edirne
*Nerium oleander* L.	13391	Dane & Öztürk	18 July 2008	Söğütlük, Edirne
*Saponaria officinalis* L.	13392	Dane & Öztürk	16 July 2008	Söğütlük, Edirne
*Tribulus terrestris* L.	13393	Dane & Öztürk	18 July 2008	Centre, Edirne

At least five leaf and stem samples were collected from each species in the budding season, and they were fixed in carnoy fluid (3:1 v/v, ethyl alcohol: acetic acid) at room temperature overnight and transferred to 70% ethyl alcohol. For light microscopy study, cross sections were obtained by hand from fixed samples and sections were treated with a solution of 2.5% sodium hypo chloride (bleaching agent) for 4 hours (Ilarslan et al. 2001). The cleared samples were covered with glycerine-gelatine to form a permanent preparation. Sections were examined under light photomicroscope (Olympus) with bright and polarized light, and the types and locations of the crystals were determined. Moreover, diameters of the druse crystals were measured using Image-Pro Plus program, and averages and standard deviations of data were calculated. Photomicrographs were taken with an Olympus digital camera and processed in Adobe PhotoShop CS2 version 9.

## Results

In the present study, six of the nine examined plants were found to contain calcium oxalate crystals. Types of crystals found in the leaves and stems of examined plants were given in Table 2. Three types of calcium oxalate crystals were observed: druses, prismatic crystals and crystal sands. Types and distribution of crystals in each of the studied plant are described in details below.

**Table 2:** **Comparison of the types of calcium oxalate crystals and content of the toxic substance found in plant organs**

Species	Types of crystals in leaves	Types of crystals in stems	Content of toxic substances	Poisonous organs
***Cynanchum acutum*** **L.**	Druse and prismatic	Druse	Vincetoksin glycoside (Öztürk et al. 2008)	Entire plant (Öztürk et al. 2008)
***Tribulus terrestris*** **L.**	Druse	Druse	Three sapogenins (Diosgenin, ruscogenin, gitogenin) (Fuller and McClintock 1986)	Entire Plant (Fuller and McClintock 1986)
***Hedera helix*** **L.**	Druse	Druse	Hederin glycoside, Hederagenin, and other triterpene saponins, sesquiterpenes, falcarinol (Fuller and McClintock 1986; Wink 2009)	Entire plant, especially leaves and fruits (Wink 2009)
***Saponaria officinalis*** **L.**	Druse	Druse	Saponin Glycoside (triterpene saponins) (Wink 2009)	Entire plant especially seeds (Muca et al. 2012)
***Humulus lupulus*** **L.**	Druse	Druses and Crystal sands	Non poisonous (Baytop 1999)	Non poisonous (Baytop 1999)
***Nerium oleander*** **L.**	Druse	Druse and Prismatic	Oleandrin glycoside and several other cardenolides (Wink 2009)	Entire plant (Wink 2009)
***Hypericum perforatum*** **L.**	Absent	Absent	Hypericin , Glyco-Alkaloid (Fuller and McClintock 1986)	Entire plant, especially leaves and flowers (Fuller and McClintock 1986)
***Aristolochia clematitis*** **L.**	Absent	Absent	Aristolochic acid and related alkaloids, aristolohin alkaloid magnoflorine (Wink 2009)	Entire plant (Wink 2009)
***Chelidonium majus*** **L.**	Absent	Absent	Chelidonine, sanguinarine, berberine and other isoquinoline alkaloids (Wink 2009)	All parts (Wink 2009)

### *Cynanchum acutum* L.

**Figure 1:**
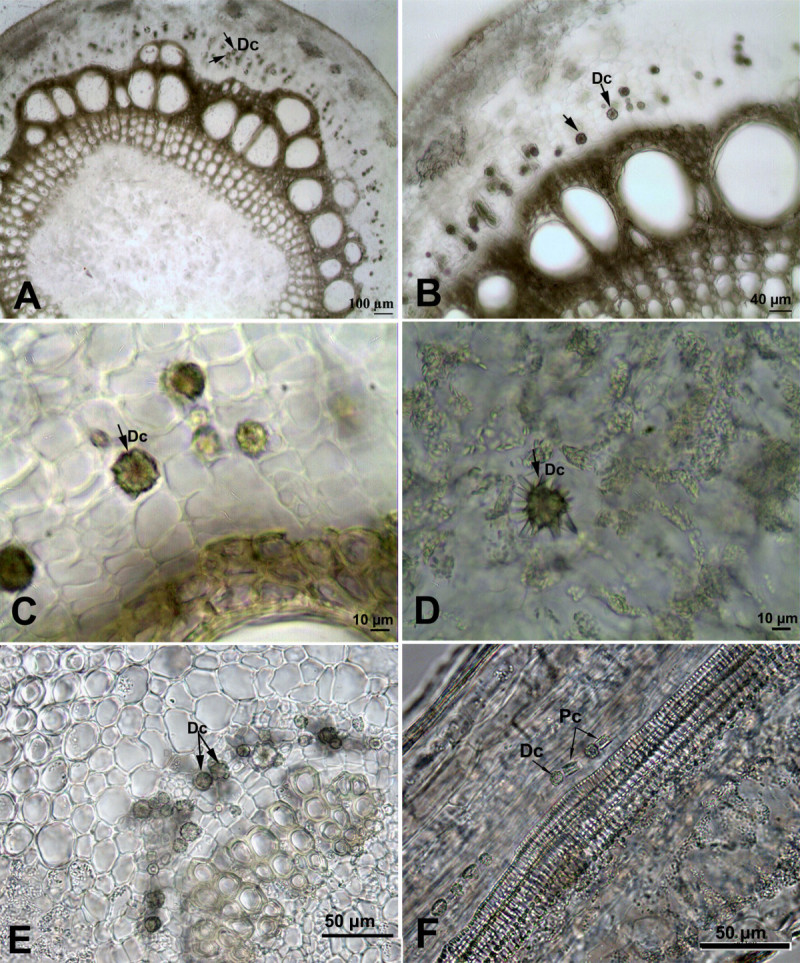
**Calcium oxalate crystals in the stem and leaf of** ***Cynanchum acutum*** **. (A-C)** Druse crystals in the cortex of the stem at different magnifications. **(D)** Druse crystals in the leaf mesophyll. **(E)** Druse crystals around the major vein. **(F)** Druse and prismatic crystals along vascular bundle. (Dc: Druse crystals; Pc: Prismatic crystals).

Druse crystals were observed in the stem and leaves of *Cynanchum acutum*. They were numerous in the stem cortex (12.5 ± 1.93 μm in diameter) of the *Cynanchum acutum*, but were scarce in its leaves (Figure 1). In the leaves, druse crystals were distributed either around the midrib or along the minor veins near xylem vessels. Around major vein, druse crystals in different sizes (9.5 ± 2.2 μm in diameter) were in clusters (Figure 1E). However, along minor veins a few small druse crystals (8 μm in diameter) and prismatic crystals having conspicuous cores were observed (Figure 1F).

### *Tribulus terrestris* L.

**Figure 2:**
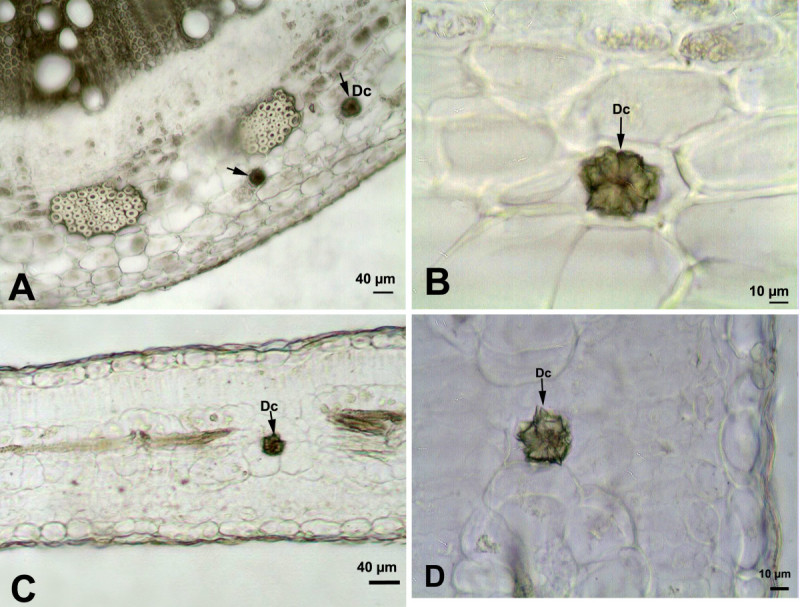
**Druse crystals in the stem and leaf of** ***Tribulus terrestris.*** **(A, B)** Druse crystals in the cortex of the stem. **(C, D)** Druse crystal in the leaf mesophyll cells. (Dc: Druse crystals).

In *Tribulus terrestris,* druse crystals were sparsely distributed in the mesophyll tissue of the leaf (24.5 ± 3 μm in diameter) and in the cortex tissue (22.2 ± 4 μm in diameter) of the stem (Figure 2).

### *Hedera helix* L.

**Figure 3:**
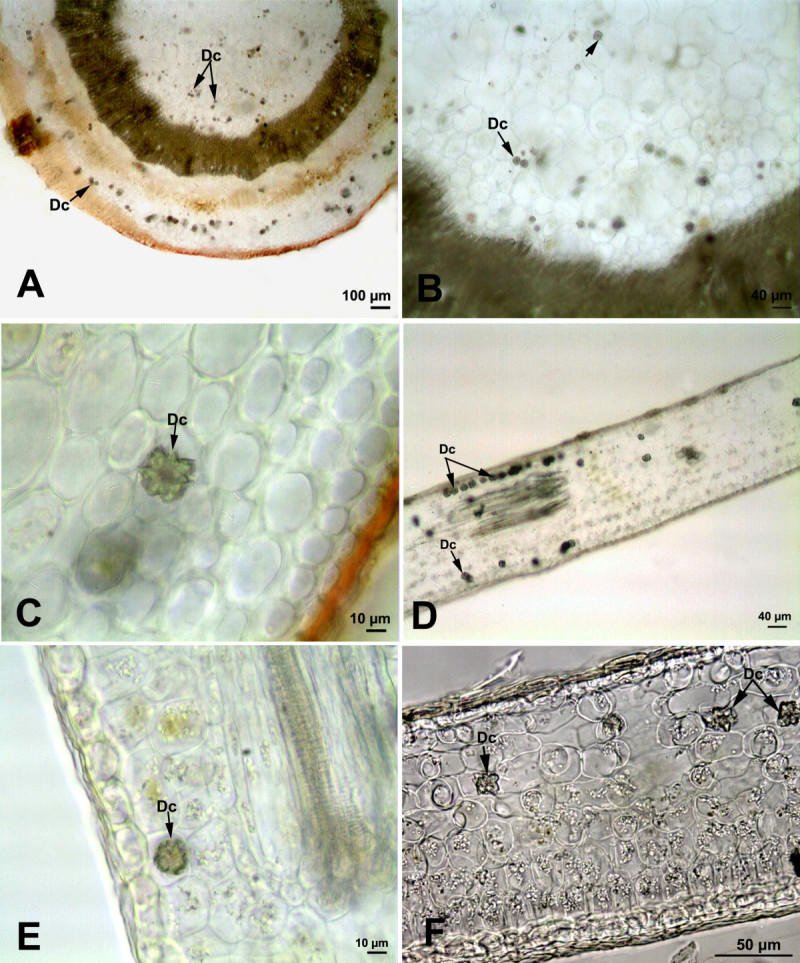
**Druse crystals in the stem and leaf of** ***Hedera helix*** **(arrow). (A)** Druse crystals in the cortex and pith cells of the stem. **(B)** Druse crystals in the pith cells of the stem. **(C)** Druse crystal in the cortex cells of the stem. **(D-F)** Distribution of druse crystals in leaf mesophyll cells. (Dc: Druse crystals).

Druse crystals were identified in the stem and leaves of *Hedera helix* (Figure 3). In the stem, druse crystals were observed both in the cortex and pith tissues (Figure 3A-C). In the cortex tissue, moderate number of druse crystals (20.6 ± 1.5 μm in diameter) were dispersed either singly or in groups of two or three. However, in pith tissue druse crystals (17.1 ± 5 μm in diameter) were rare.

In the leaf, druse crystals (16.2 ± 3.9 μm in diameter) were observed both in the mesophyll tissue and around the vascular bundles (Figure 3D-F). In the mesophyll tissue, they were densely distributed in the spongy tissue. However, only a few druse crystals were present in the palisade tissue. About 9–10 crystals which arranged in one row of cells were observed along some regions of minor veins. Additionally, some of the druse crystals were in contact with the xylem or phloem vessels.

### *Saponaria officinalis* L.

**Figure 4:**
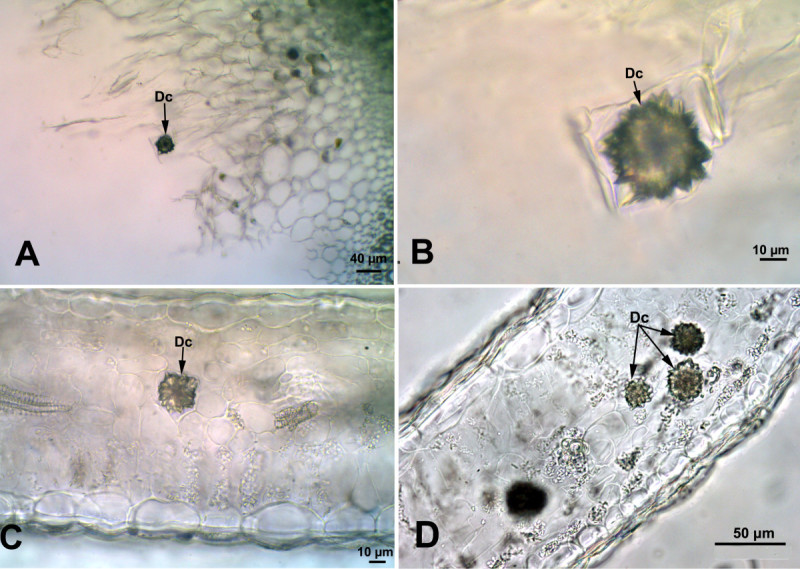
**Druse crystals in the stem and leaf cross sections of** ***Saponaria officinalis*** **. (A, B)** Druse crystals in the pith cells of the stem. **(C, D)** Distribution of the druse crystals in leaf mesophyll cells. (Dc: Druse crystals).

A few druse crystals were observed in the pith tissue of the stem (26 ± 1.2 μm in diameter) and mesophyll tissue of the leaf (Figure 4). In the leaf, druse crystals of different sizes (22 ± 4.2 μm in diameter) were distributed rarely in the spongy mesophyll.

### *Humulus lupulus* L.

**Figure 5:**
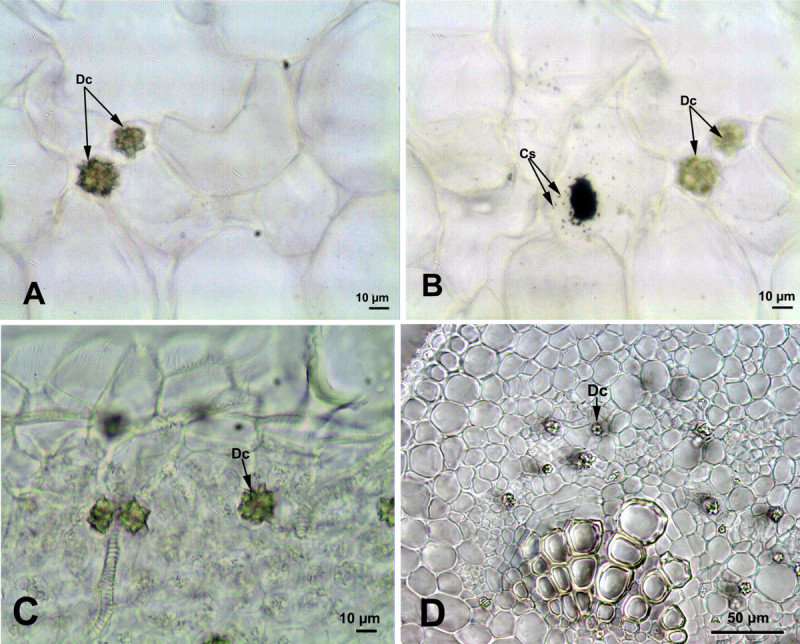
**Druse crystals in the stem and leaf cross sections of** ***Humulus lupulus*** **. (A)** Druse crystals in the pith cells of the stem. **(B)** Druses and crystal sands in the pith cells of the stem. **(C)** Druse crystal in leaf mesophyll cells. **(D)** Druse crystals around the vascular bundle (Dc: Druse crystals; Cs: Crystal sands).

A few druse crystals (16.6 ± 1 μm in diameter) and crystal sands were identified in the pith tissue of the stem of *Humulus lupulus* (Figure 5A, B). In the leaves, druse crystals were located both around the vascular tissue and in the palisade and spongy tissue of the mesophyll (Figure 5C, D). In the mesophyll tissue, druses were rare and their diameters changed between 12.5 – 16.4 μm (14.3 ± 2 μm). However, around vascular bundle, a cluster of druse crystals at various sizes (11–20 μm in diameter) was observed (Figure 5D). Druse crystals that were located near the vascular bundle were smaller than the others.

### *Nerium oleander* L.

**Figure 6:**
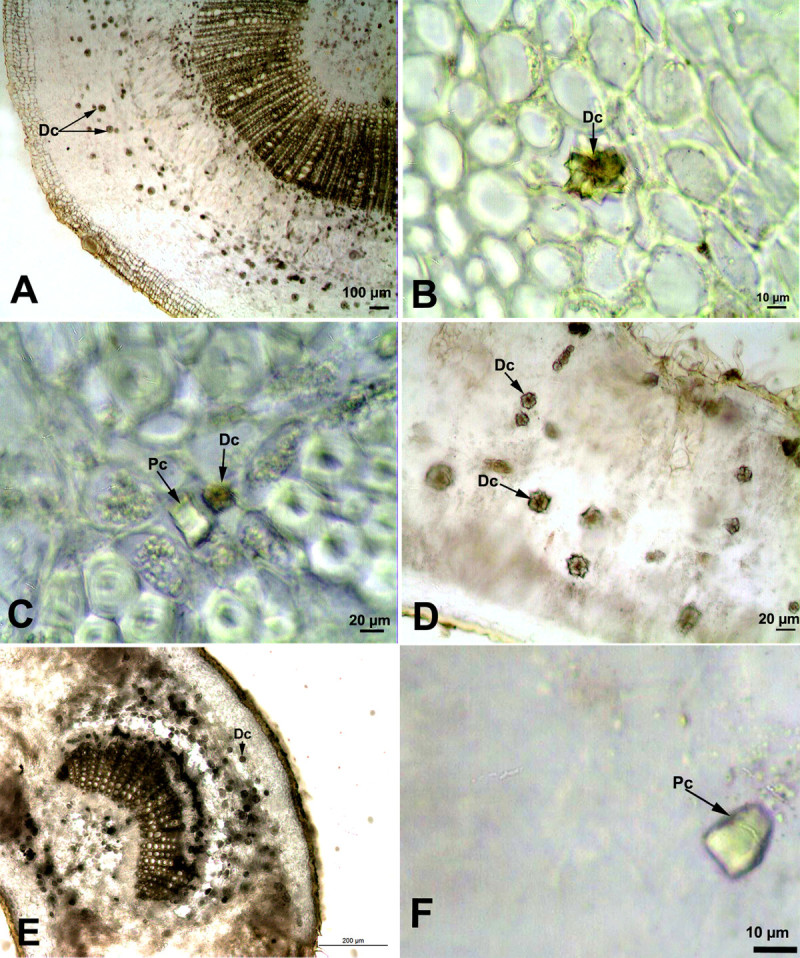
**Crystals in the stem and leaf cross-sections of** ***Nerium oleander*** **. (A, B)** Druse crystals in the cortex of the stem, **(C)** Druse and prismatic crystals in the cortex of the stem, **(D)** Druse crystals in leaf mesophyll cells, **(E)** Druse crystals around the midrib, **(F)** Prismatic crystals in leaf mesophyll cells. (Dc: Druse crystals; Pc: Prismatic crystals).

In *Nerium oleander*, numerous single druse crystals were identified in the cortex (17.5 ± 2.86 μm in diameter) and pith tissues (18.5 ± 2.5 μm in diameter) of the stem (Figure 6A, B). In the leaves, druse crystals were observed both around the major vein (19.1 ± 2.8 μm in diameter) and in the mesophyll tissue (22.6 ± 6 μm in diameter). In the mesophyll tissue, druse crystals were distributed either singly or 3–4 crystals were arranged in one row of cells (Figure 6D). In addition to druses, a few prismatic crystals were also observed in the cortex tissue of the stem (Figure 6C) and leaf mesophyll cells of *Nerium oleander* (Figure 6F).

No crystals were observed in the leaves and stems of *Aristolochia clematitis, Chelidonium majus* and *Hypericum perforatum.*

## Discussion and conclusion

Although numerous studies have been made on calcium oxalate crystals, only a few of them (Fasset 1973 Genua and Hillson 1985 Doaigey 1991) have investigated the calcium oxalate crystals in toxic plant organs. Genua and Hillson (1985), in their study of oxalate crystals in toxic plant organs, concluded that although the relationship between druses and the poisonous properties of the plant was not fully known, druses may be the main mechanical irritants in toxic plants. Besides this, Doaigey (1991) has tried to correlate toxic parts of the plant with the distribution of calcium oxalate crystals; however, he has concluded that there is no absolute relationship between calcium oxalate crystals and the poisonous properties of the plants.

In the present study, *Humulus lupulus* which was reported as non-toxic (Baytop 1999) examined as a reference and it was found that it contains druse crystals in the mesophyll cells of its leaves and druses and crystal sands in the pith cells of its stem. Wu and Kuo-Huang (1997) have studied calcium crystals in the leaves of *Humulus scandens* and they have reported only calcium carbonate crystals in its leaves. The differences in the types of calcium crystals between these two different species of the genus *Humulus* is not surprising. Because, although crystal formation in plants is mostly under genetic control (Ilarslan et al. 2001), the location, size and other properties of crystals may be effected by physical, chemical and biological conditions such as light, temperature, pH, ion concentration and herbivory (Franceschi and Horner 1980; Molano-Flores 2001; Kuo-Huang et al. 2007). Moreover, features of calcium crystals in the leaves of *Humulu* s may be useful in intra genus classification.

The stem and leaves of *Cynanchum acutum* and *Hedera helix* were reported as poisonous (Fuller and McClintock 1986) and were found to have druse crystals. The whole of *Hypericum perforatum* (Baytop 1963,1989; Fuller and McClintock 1986), *Nerium oleander* (Baytop 1989; Wink 2009; Muca et al. 2012), *Tribulus terrestris* (Fuller and McClintock 1986), *Saponaria officinalis* (Muca et al. 2012), *Aristolochia clematitis* (Wink 2009) and *Chelidonium majus* (Wink 2009) display toxic properties but only stem and leaves of *Nerium oleander* have druses and prismatic crystals. Additionally, druses have been observed both in the stem and leaves of *Tribulus terrestris* and *Saponaria officinalis.* On the other hand, calcium oxalate crystals were not identified in the stem and leaves of *Aristolochia clematitis*, *Chelidonium majus* and *Hypericum perforatum* (Table 2).

In the present study, although druse crystals have been observed both in the stem and leaves of *Saponaria officinalis*, in the study of Ataşlar (2004) on *Saponaria kotschyi* Boiss, druses have been reported only in the leaves and roots of *Saponaria kotschyi* but not in the stem. So this supports the idea that location of the crystals within a taxon is often very specific and may be represented as a taxonomic character (Genua and Hillson 1985; Prychid and Rudall 1999; Lersten and Horner 2000).

In *Nerium oleander* druse and prismatic crystals were observed in the adjacent cells of the cortex tissue of the stem. This confirms the idea of Scurfield et al. (1973) which suggested that impurities which would most likely differ from cell to cell, may lead to the formation of different crystal types in the same tissue. However, this result appears to be inconsistent with the fact that some plants contain only one type of crystal through the many tissues (Franceschi and Horner 1980). Similar to our results, Doaigey (1991) have also reported druses and prismatic crystals in the leaves and stem of *Nerium oleander* growing naturally in Saudi Arabia.

Poisonous compounds in the studied species are either alkaloids or glycosides. Five of the eight poisonous plant species contain glycosidic compounds, and have druse crystals. However, three of the studied plants have alkaloids as poisonous compounds, and all of them are devoid of calcium oxalate crystals (Table 2). Therefore, the results of the present study point out the correlation between the kinds of toxic substance and the presence of calcium oxalate crystals in the studied plants. However, in the study of Doaigey (1991), investigating calcium oxalate crystals in the sixteen species of poisonous plants, calcium oxalate crystals have been reported in the four of the seven species having alkaloids and in the three of the seven species containing glycosidic compounds. The results of the Doaigey’s study don’t confirm the findings of our study related with alkaloids and glycosides. Consequently there is no sufficient evidence to prove the relationship between the kind of toxic substances and occurrence, types and distribution of calcium oxalate crystals in plant organs.

In the light of the results, collected data and literature survey it can be concluded that there is no absolute correlation between the presence of calcium oxalate crystals and the toxicity of plant organs. This conclusion confirms the results of the studies which are carried out by Genua and Hillson (1985), and Doaigey (1991). Furthermore, druses are the most abundant calcium oxalate crystals in poisonous organs of the studied plants. Hence, as stated by Genua and Hillson (1985) druses may function as main mechanical irritants in toxic plants.

## Authors’ original submitted files for images

Below are the links to the authors’ original submitted files for images. Authors’ original file for figure 1 Authors’ original file for figure 2 Authors’ original file for figure 3 Authors’ original file for figure 4 Authors’ original file for figure 5 Authors’ original file for figure 6

## References

[CR1] Aplin TEH (1976). Poisonous Garden Plants and Other Plants Harmful to Man in Australia. Bulletin 3964.

[CR2] Ataşlar E (2004). Morphological and anatomical investigations on the *Saponaria kotschyi* Boiss. (Caryophyllaceae). Turk J Bot.

[CR3] Aybeke M (2012a). Comparative anatomy of selected rhizomatous and tuberous taxa of subfamilies Orchidoideae and Epidendroideae (Orchidaceae) as an aid to identification. Plant Syst Evol.

[CR4] Aybeke M (2012b). Anther wall and pollen development in *Ophrys mammosa* L. (Orchidaceae). Plant Syst Evol.

[CR5] Aybeke M, Sezik E, Olgun G (2010). Vegetative anatomy of some *Ophrys*, *Orchis* and *Dactylorhiza* (Orchidaceae) taxa in Trakya region of Turkey. Flora.

[CR6] Baytop T (1963). Türkiye’nin tıbbi ve zehirli bitkileri.

[CR7] Baytop T (1989). Türkiye’de zehirli bitkiler, bitki zehirlenmeleri ve tedavi yöntemleri.

[CR8] Baytop T (1999). Türkiye’de tıbbi bitkiler ile tedavi.

[CR9] Buttrose MS, Lott JNA (1978). Calcium oxalate druse crystals and other inclusions in seed protein bodies: Eucalyptus and jojoba. Can J Bot.

[CR10] Çalışkan M (2000). The metabolism of oxalic acid. Turk J Zool.

[CR11] Cheeke PR (1995). Endogenous toxins and mycotoxins in forage grasses and their effects on livestock. J Anim Sci.

[CR12] Dane F, Hüseyinova G, Meriç Ç (2000). Some ultrastructural observation on calcium oxalate raphide crystal idioblasts and meristematic cells of the adventive root tip of *Sternbergia lutea* (L.) Ker- Gawl. Ex Sprengel (Amaryllidaceae). Turk J Bot.

[CR13] Dhillon KS, Paul BS, Bajwa RS, Singh J (1971). A preliminary report on a peculiar type of napiergrass (*Pennisetum purpureum*, 'Pusa giant’) poisoning in buffalo calves. Indian J Anim Sci.

[CR14] Doaigey AR (1991). Occurrence, type, and location of calcium oxalate crystal in leaves and stems of 16 species of poisonous plants. Am J Bot.

[CR15] Ekici N, Dane F (2007). Calcium oxalate crystals in floral organs of *Galanthus* sp. (Amaryllidaceae). Asian J Plant Sci.

[CR16] Ekici N, Dane F (2009). Calcium oxalate crystals during development of male and female gametophyte in *Leucojum aestivum* (Amaryllidaceae). Jabs.

[CR17] Faheed F, Mazen A, Abd Elmohsen S (2013). Physiological and ultrastructural studies on calcium oxalate crystal formation in some plants. Turk J Bot.

[CR18] Fasset DW (1973). Oxalates. Toxicants Occurring Naturally in Foods.

[CR19] Franceschi VR (1989). Calcium oxalate formation is a rapid and reversible process in *Lemna minor*. Protoplasma.

[CR20] Franceschi VR, Horner HT (1980). Calcium oxalate crystals in plants. Bot Rev.

[CR21] Frohne D, Pfander J (1984). A Colour Atlas of Poisonous Plants.

[CR22] Fuller TC, McClintock E (1986). Poisonous Plants of California.

[CR23] Genua JM, Hillson CJ (1985). The occurrence, type and location of calcium oxalate crystals in the leaves of fourteen species of Araceae. Ann Bot.

[CR24] Grimson MJ, Arnott HJ (1983). An ultrastructural study of druse crystals in the abscission zone of *Phyllanthus niruri* L. Scan Electron Microsc.

[CR25] Horner HT, Wagner BL (1980). The association of druse crystals with the developing stomium of *Capsicum annuum* (Solanaceae) anthers. Am J Bot.

[CR26] Horner HT, Wagner BL (1992). Association of four different calcium crystals in the anther connective tissue and hypodermal stomium of *Capsicum annuum* (Solanaceae) during microsporogenesis. Am J Bot.

[CR27] Horner HT, Wagner BL, Khan SR (1995). Calcium oxalate formation in higher plants. Calcium oxalate in biological systems.

[CR28] Horner HT, Whitmoyer RE (1972). Raphide crystal cell development in leaves of *Psychotria punctata* (Rubiaceae). J Cell Sci.

[CR29] Horner HT, Kausch AP, Wagner BL (2000). Ascorbic acid: a precursor of oxalate in crystal idioblasts of *Yucca torrey* in liquid root culture. Int J Plant Sci.

[CR30] Ilarslan H, Palmer RG, Horner HT (2001). Calcium oxalate crystals in developing seeds of soybean. Ann Bot.

[CR31] Kinzel H (1989). Calcium in the vacuoles and cell walls of plant tissues. Flora.

[CR32] Kuo-Huang LL, Ku MSB, Franceschi VR (2007). Correlations between calcium oxalate crystals and photosynthetic activities in palisade cells of shade-adapted *Peperomia glabella*. Bot Stud.

[CR33] Lersten NR, Horner HT (2000). Calcium oxalate crystals types and trends in their distribution patterns in leaves of *Prunus* (Rosaceae: Prunoideae). Plant Syst Evol.

[CR34] Lersten NR, Horner HT (2006). Crystal macropattern development in *Prunus serotina (* Rosaceae, Prunoideae) leaves. Ann Bot.

[CR35] Lott JNA, Buttrose MS (1978). Location of reserves of mineral elements in seed protein bodies: macadamia nut, walnut, and hazel nut. Can J Bot.

[CR36] McNair JB (1932). The intersection between substances in plants: essential oils and resins, cyanogen and oxalate. Am J Bot.

[CR37] Meriç Ç (2008). Calcium oxalate crystals in *Conyza canadensis* (L.) Cronq. and *Conyza* b*onariensis* (L.) Cronq. (Asteraceae: Astereae). Acta Biol Szeged.

[CR38] Meriç Ç (2009). Calcium oxalate crystals in some species of the Tribe Inuleae (Asteraceae). Acta Biol Cracov Ser Bot.

[CR39] Meriç Ç, Dane F (2004). Calcium oxalate crystals in floral organs of *Helianthus annuus* L. and *tuberosus* L. (Asteraceae). Acta Biol Szeged.

[CR40] Molano-Flores B (2001). Herbivory and calcium concentrations affect calcium oxalate crystal formation in leaves of *Sida* (Malvaceae). Ann Bot.

[CR41] Muca B, Yıldırım B, Özçelik Ş, Koca A (2012). Isparta’s (Turkey) poisonous plants of public access places. BioDiCon.

[CR42] Öztürk M, Uysal İ, Gücel S, Mert T, Akçiçek E, Sezgin Ç (2008). Ethnoecology of poisonous plants of Turkey and Northern Cyprus. Pak J Bot.

[CR43] Prychid CJ, Rudall PJ (1999). Calcium oxalate crystals in monocotyledons: a review of their structure and systematics. Ann Bot.

[CR44] Rahman MM, Kawamura O (2011). Oxalate accumulation in forage plants: some agronomic, climatic and genetic aspects. Asian Aust J Anim Sci.

[CR45] Rahman MM, Niimi YI, Kawamura O (2006). Effects of seasons, variety and botanical fractions on oxalate content of napiergrass (*Pennisetum purpureum* Schumach). Grassl Sci.

[CR46] Rudall PJ, Chase MW (1996). Systematics of Xanthorrhoeaceae *sensu lato*: evidence for polyphyly. Telopea.

[CR47] Rupali T, Chavan S, Pandhure N (2012). Occurrence of chloride enriched calcium oxalate crystal in *cissus quadrangularis* linn. Int J Pharm.

[CR48] Scurfield G, Michell AJ, Silva SR (1973). Crystals in woody stems. Bot J Linn Soc.

[CR49] Seçmen O, Leblebici E (1987). Yurdumuzun Zehirli Bitkileri.

[CR50] Sutherland JM, Sprent JI (1984). Calcium-oxalate crystals and crystal cells in determinate root nodules of legumes. Planta.

[CR51] Tilton VR, Horner HT (1980). Calcium oxalate raphide crystals and crystalliferous idioblasts in the carpels of *Ornithogalum caudatum*. Ann Bot.

[CR52] Webb MA (1999). Cell-mediated crystallization of calcium oxalate in plants. Plant Cell.

[CR53] Webb MA, Arnott HJ (1982). A survey of calcium oxalate crystals and other mineral inclusions in seeds. Scan Electron Micros.

[CR54] Webb MA, Arnott HJ (1983). Inside plant crystals: a study of the noncrystalline core in druses of *Vitis vinifera* endosperm. Scan Electron Micros.

[CR55] Wink M (2009). Mode of action and toxicology of plant toxins and poisonous plants.

[CR56] Wu CC, Kuo-Huang LL (1997). Calcium crystals in the leaves of some species of Moraceae. Bot Bull Acad Sin.

