# Supplementary figures and images for: Occurrence, types and distribution of calcium oxalate crystals in leaves and stems of some species of poisonous plants
Source: Bot Stud. 2014 Mar 15;55:32. doi: 10.1186/1999-3110-55-32 (PMC5432768; doi:10.1186/1999-3110-55-32)

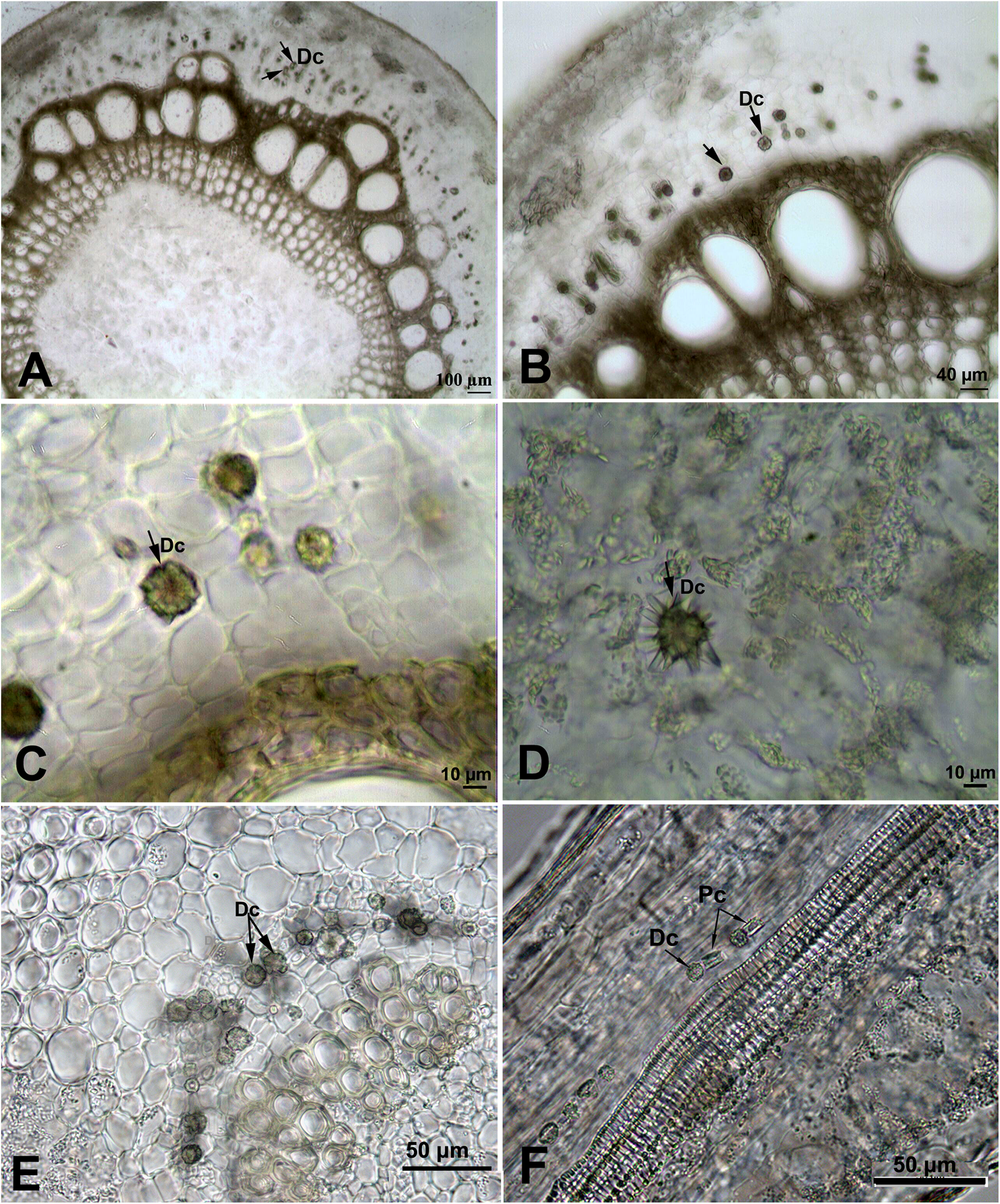

Supplement: Supplementary file 1 — Authors’ original file for figure 1 [file 40529_2014_88_MOESM1_ESM.tif]

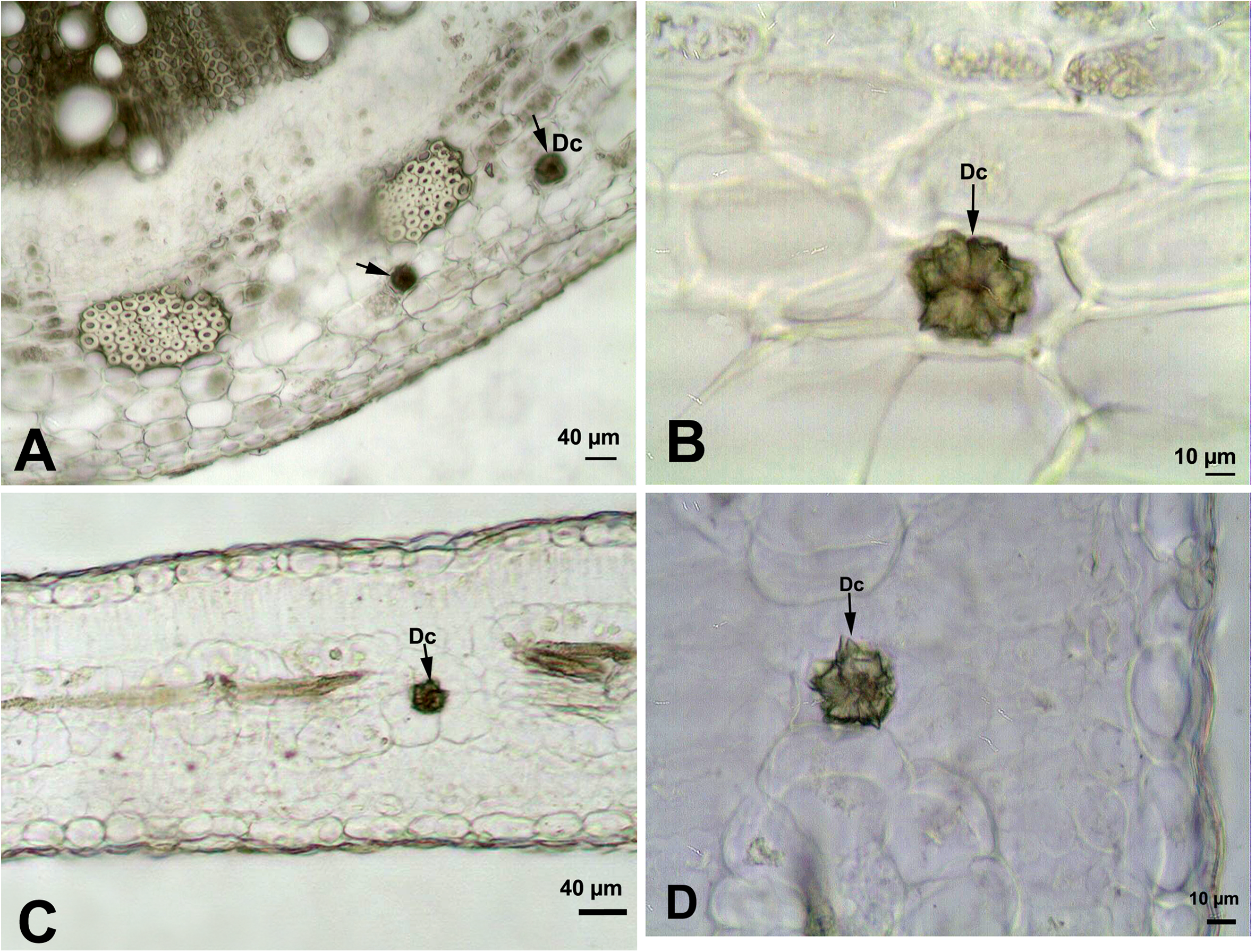

Supplement: Supplementary file 2 — Authors’ original file for figure 2 [file 40529_2014_88_MOESM2_ESM.tif]

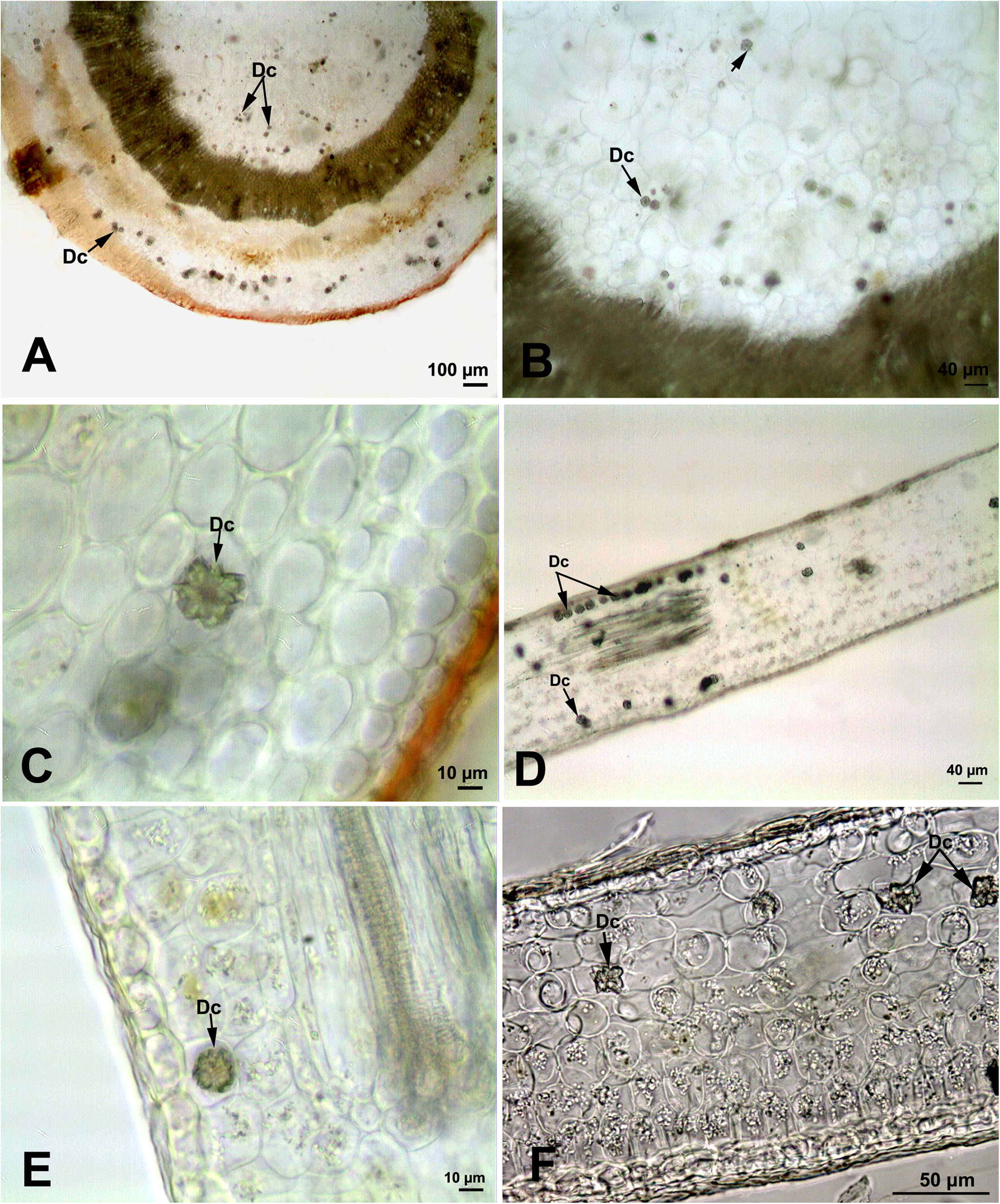

Supplement: Supplementary file 3 — Authors’ original file for figure 3 [file 40529_2014_88_MOESM3_ESM.tif]

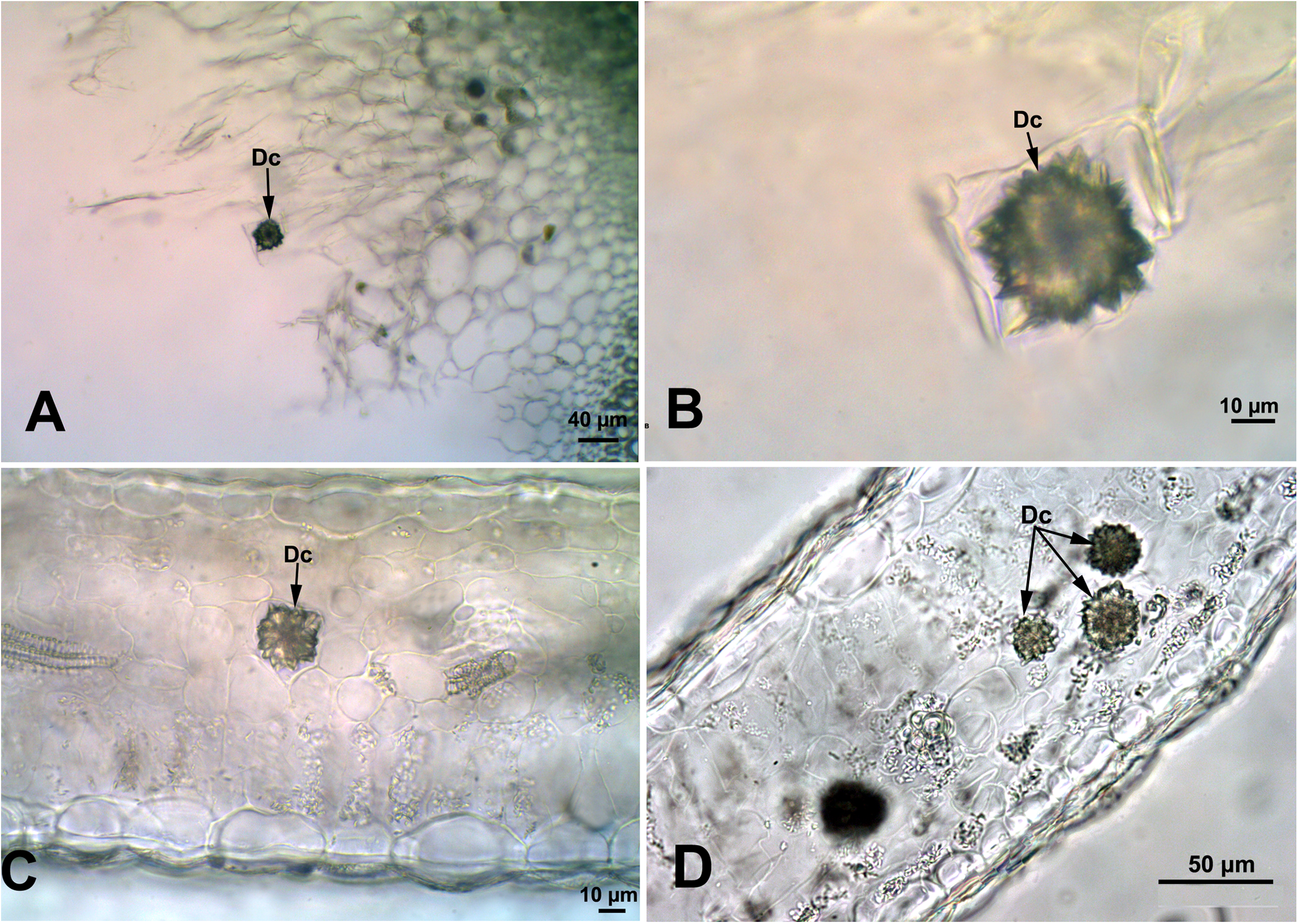

Supplement: Supplementary file 4 — Authors’ original file for figure 4 [file 40529_2014_88_MOESM4_ESM.tif]

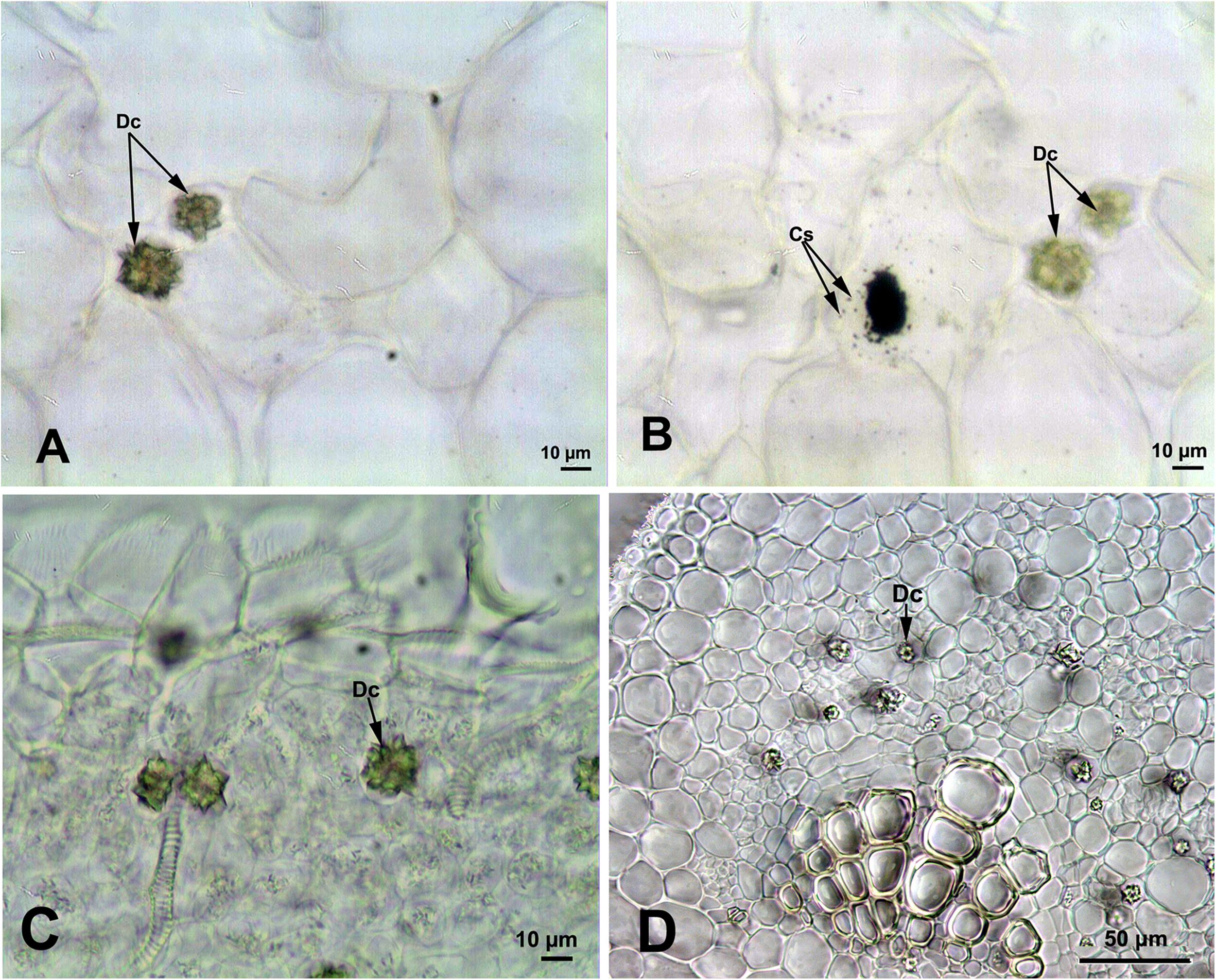

Supplement: Supplementary file 5 — Authors’ original file for figure 5 [file 40529_2014_88_MOESM5_ESM.tif]

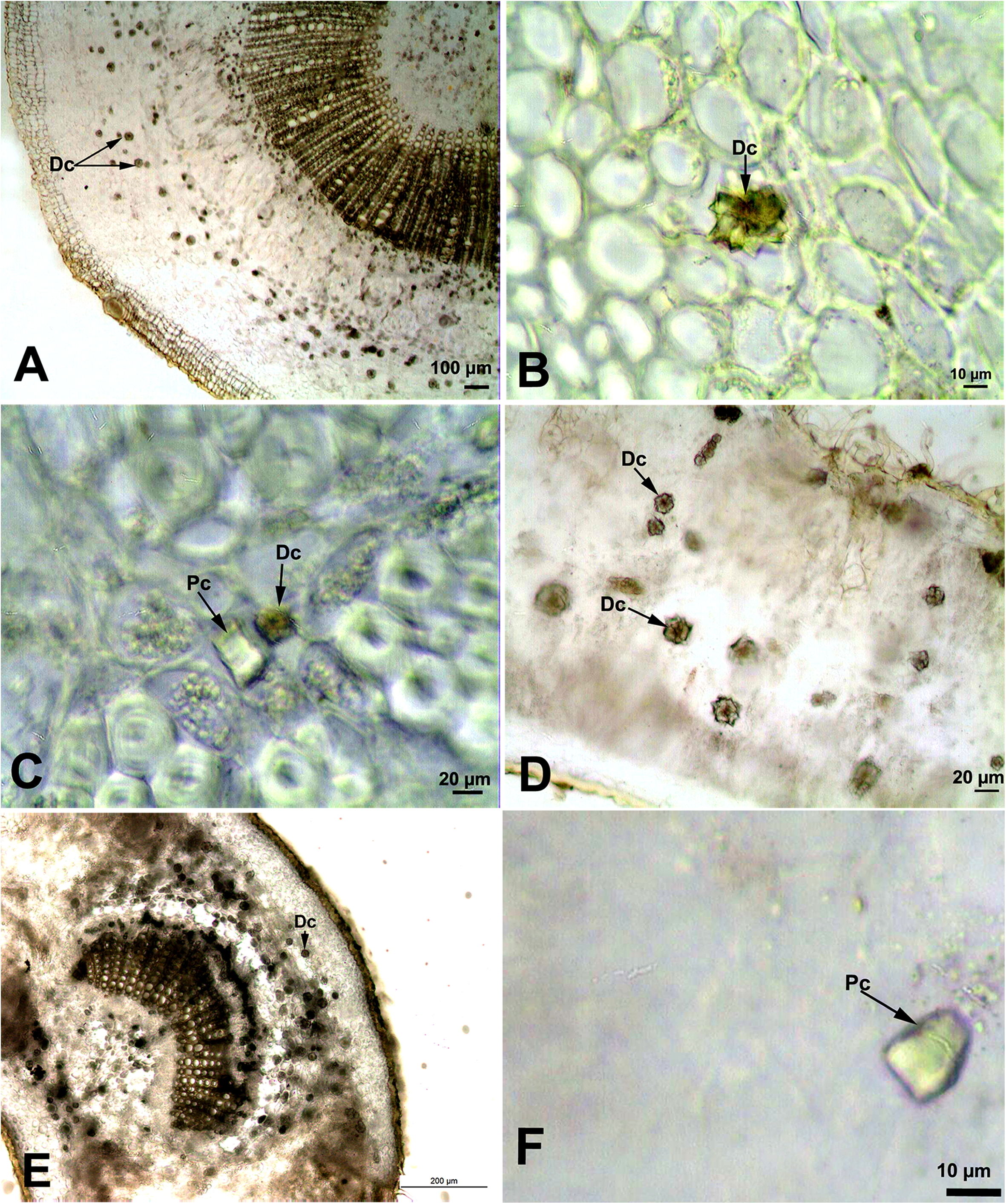

Supplement: Supplementary file 6 — Authors’ original file for figure 6 [file 40529_2014_88_MOESM6_ESM.tif]
